# MR-Less Surface-Based Amyloid Assessment Based on ^11^C PiB PET

**DOI:** 10.1371/journal.pone.0084777

**Published:** 2014-01-10

**Authors:** Luping Zhou, Olivier Salvado, Vincent Dore, Pierrick Bourgeat, Parnesh Raniga, S. Lance Macaulay, David Ames, Colin L. Masters, Kathryn A. Ellis, Victor L. Villemagne, Christopher C. Rowe, Jurgen Fripp, AIBL Research Group

**Affiliations:** 1 CSIRO Preventative Health Flagship, CSIRO Computational Informatics, The Australian e-Health Research Centre, Herston, Australia; 2 Department of Nuclear Medicine and Centre for PET, Austin Hospital, Heidelberg, Australia; 3 Department of Computer Science and Software Engineering, University of Wollongong, Wollongong, Australia; 4 CSIRO Preventative-Health National Research Flagship, Parkville, Australia; 5 Mental Health Research Institute/Florey Institute of Neuroscience and Mental Health, The University of Melbourne, Parkville, Australia; 6 National Ageing Research Institute, Parkville, Australia; 7 Academic Unit for Psychiatry of Old Age, Department of Psychiatry, The University of Melbourne, Kew, Parkville, Australia; University of Manchester, United Kingdom

## Abstract

**Background:**

β-amyloid (Aβ) plaques in brain's grey matter (GM) are one of the pathological hallmarks of Alzheimer's disease (AD), and can be imaged in vivo using Positron Emission Tomography (PET) with ^11^C or ^18^F radiotracers. Estimating Aβ burden in cortical GM has been shown to improve diagnosis and monitoring of AD. However, lacking structural information in PET images requires such assessments to be performed with anatomical MRI scans, which may not be available at different clinical settings or being contraindicated for particular reasons. This study aimed to develop an MR-less Aβ imaging quantification method that requires only PET images for reliable Aβ burden estimations.

**Materials and Methods:**

The proposed method has been developed using a multi-atlas based approach on ^11^C-PiB scans from 143 subjects (75 PiB+ and 68 PiB- subjects) in AIBL study. A subset of 20 subjects (PET and MRI) were used as atlases: 1) MRI images were co-registered with tissue segmentation; 2) 3D surface at the GM-WM interfacing was extracted and registered to a canonical space; 3) Mean PiB retention within GM was estimated and mapped to the surface. For other participants, each atlas PET image (and surface) was registered to the subject's PET image for PiB estimation within GM. The results are combined by subject-specific atlas selection and Bayesian fusion to generate estimated surface values.

**Results:**

All PiB+ subjects (N = 75) were highly correlated between the MR-dependent and the PET-only methods with Intraclass Correlation (ICC) of 0.94, and an average relative difference error of 13% (or 0.23 SUVR) per surface vertex. All PiB- subjects (N = 68) revealed visually akin patterns with a relative difference error of 16% (or 0.19 SUVR) per surface vertex.

**Conclusion:**

The demonstrated accuracy suggests that the proposed method could be an effective clinical inspection tool for Aβ imaging scans when MRI images are unavailable.

## Introduction

β-amyloid (Aβ) plaques are one of the neuropathological hallmarks of Alzheimer's disease (AD), which starts accumulating several years before the clinical phenotype of dementia is manifested [5], [32]–[33]. The development of molecular imaging agents allows assessing Aβ deposition in vivo. The most widely used Aβ imaging radiotracer is Pittsburgh Compound B (^11^C-PiB), which binds with high affinity and high specificity to Aβ plaques [15]. It has been shown that AD patients tend to have 50% to 90% higher PiB retention than age-matched normal controls in cortical brain regions such as frontal, precuneus, parietal and temporal cortices [40], [26], [20], [8]. High PiB retention has also been found in mild cognitive impairment (MCI) [14], and faster MCI converters had higher PiB retention than slower converters [21]. The main brain tissues: grey matter (GM), white matter (WM), and cerebrospinal fluid (CSF) have different degrees of PiB retention. For example, cortical GM has marked PiB retention in AD patients, while a much faster clearance is observed in normal controls. On the other hand WM has non-specific retention but a much slower clearance in both AD and normal controls [15], [9].

Recent research [39] indicated that the spatial pattern of amyloid deposition is related to cognitive performance and may be more informative than the biomarker of total amyloid burden. Significantly increased PiB uptake in AD patients in the middle frontal gyrus, posterior cingulated cortex and inferior parietal lobe were also found good discriminators for differentiating AD patients from normal controls [17]. Therefore, the visualization of spatial patterns of PiB uptake would be a valuable clinical tool. The cortical surface based visualization could provide a more compact representation of PiB uptake than the traditional image based visualization, which is more convenient for instant inspections by clinicians. Similar tools are widely used to assess glucose metabolism with Fluorodeoxyglucose (FDG-PET), and a good example is Neurostat (NeuroStat/3D-SSP: http://128.95.65.28/_Download). These tools benefit from the fact that FDG uptake in the WM is very low compared to that of the GM, and therefore a straightforward sum or maximum projection is sufficient to estimate the GM uptake on the cortical surface. This assumption does not hold for most Aβ imaging radiotracers, including PiB, which have significant retention in the WM, and therefore the 3D surface separating WM and GM is not well-defined and has to be estimated. This problem is compounded by the observations that individuals with low PiB retention show a lower signal in the GM than in the WM as opposed to individuals with high PiB retention where the GM signal is higher than the one in the WM. Those issues are very challenging as PET imaging lacks anatomical details and resolution to distinguish the GM/WM interface. As a result, Magnetic Resonance Imaging (MRI) is often used for the correct sampling of GM regions [38], [10], [13]. This involves segmenting the brain into GM and WM masks, and extracting the separating surface. This surface is then registered to the subject PET scan where GM PiB retention can be measured and displayed.

In standard clinical setups, when PET Aβ imaging scans are visually assessed, MRI scans are not always available (e.g. different system, MRI scan done later) or might be contraindicated (e.g. presence of metallic implants, claustrophobia, pathological tremor). This paper proposes a method to estimate the PiB retention on the cortical surface without the need of an MRI scan. Our proposed “PET-only” approach is compared to the standard method where MRI is available, which we refer to as the MRI-dependent method.

A PET-only method was reported in [16]. It employed a single MRI atlas with segmented tissues, co-registered to a PET atlas from the same subject. When a new subject PET was registered to the PET atlas, the MRI atlas could be aligned using the same transform to estimate the GM. The maximal PiB retention within the subject GM was measured along the normal direction of the brain surface. The selection of that single atlas remains an issue and may affect the performance of the method.

We hypothesize that using multiple atlases and a probabilistic estimation of the GM would allow PiB retention to be estimated accurately so that acquiring an additional MRI is unnecessary. We extend this concept by allowing spatially “local” atlas selection, where the selected atlases are locally combined at each brain surface location using a Bayesian framework to improve the posterior probability of the estimation. This essentially provides a localized linear weighting for each atlas. In addition, we introduce a “soft” tissue probability map to locally guide the estimation of the PiB retention. With these strategies, the PiB retention can be estimated directly from the PET image alone, without explicit segmentation of grey matter.

In this study, we do not evaluate the estimated PiB uptake to classify AD from NC as it has already been reported that the spatial pattern of grey matter PiB uptake (with the use of MRI) could be employed for classifying the risk of developing AD [17], and different classification schemes bring different classification accuracies. Such a divergence would not inform on the accuracy of our proposed PiB estimate method. Instead, we focus on validating the agreement of the proposed PET-only method with the traditional MR-dependent method. As long as the estimation discrepancy of the two methods is minimized, classification schemes that are applicable to PiB uptake estimated by the MR-dependent method can be potentially transported to that by the PET-only method.

## Materials and Methods

### Ethics Statement

All the data were obtained from the Australian Imaging Biomarkers and Lifestyle study [7]. The complete listing of AIBL investigators is available at. Core funding for the AIBL study was provided by the CSIRO (http://www.csiro.au/) Flagship Collaboration Fund and the Science and Industry Endowment Fund (SIEF http://www.sief.org.au/) in partnership with Edith Cowan University (https://www.ecu.edu.au/), Florey neurosciences and Mental Health Research institutes (http://www.florey.edu.au/), Alzheimer's Australia (http://www.fightdementia.org.au/), National Ageing Research Institute (http://www.mednwh.unimelb.edu.au/), Austin Health (http://www.austin.org.au/), CogState Ltd. (http://cogstate.com/), Hollywood Private Hospital (http://www.hollywood.ramsayhealth.com.au/), Sir Charles Gardner Hospital (http://www.scgh.health.wa.gov.au/). The AIBL study also receives funding from the National Health and Medical Research Council (http://www.nhmrc.gov.au/), the Dementia Collaborative Research Centers program (http://www.fightdementia.org.au/victoria/dementia-collaborative-research-centres-1.aspx), The McCusker Alzheimer's Research Foundation (http://alzheimers.com.au/) and Operational Infrastructure Support from the Government of Victoria (http://www.vic.gov.au/), Australia. Pfizer International (http://www.pfizer.com.au/default.aspx) has contributed financial support to AIBL to assist with analysis of blood samples and to further the AIBL research program.

### Participants

PiB scans from 143 subjects from the AIBL study were used. The AIBL dataset is available online in LONI image data archive (https://ida.loni.ucla.edu/login.jsp?project=AIBL).These subjects underwent both MR and PiB-PET scans at the Austin Hospital (Melbourne). Approval for the study was obtained from the Austin Health Human Research Ethics Committee and St Vincent's Health Research Ethics Committee, and written informed consent for participation was obtained for each subject prior to the scans.

T1-weighted MRI were obtained using the ADNI magnetization prepared rapid gradient echo protocol at 3T, with in-plane resolution 1×1 mm and 1.2 mm slice thickness. The PiB-PET scans were acquired using an Allegro PET camera (Phillips, Amsterdam, the Netherlands). Each participant was injected with 370 MBq of ^11^C-PiB, and a 30-minute acquisition in 3D mode was performed starting 40 minutes after injection (6×5-minute frames). A transmission scan was performed for attenuation correction. PET images were reconstructed using a 3D RAMLA algorithm. More description about the PET imaging was given in [26].

All 143 subjects were categorized into either PiB+ (“AD-like”) or PiB- (“Control-like”) groups according to their neocortical standard uptake value ratio (SUVR). SUVR is defined as the ratio of the regional brain PiB retention to the average of cerebellar cortex area [24], [18], [12]. In this study, the neocortical SUVR value was summarized using the mean SUVR in the GM masked neocortical region composed of the frontal, superior parietal, lateral temporal, occipital and anterior and posterior cingulated regions of the AAL ROI atlas [35]. This is consistent with [27] where similar ROIs were used for SUVR computation for the AIBL dataset. Following [27], subjects with neocortical SUVR greater than 1.5 were labeled PiB+ and those with neocortical SUVR equal or less than 1.5 were labeled PiB-. The cut-off value of 1.5 was also used by in [11], [30]. In order to get a more detailed assessment, each image group is further subdivided into those PiB- with a neocortical SUVR above and below 1.3, and those PiB+ with a neocortical SUVR above and below 2.0. The demographic information about the subjects is presented in Table 1. They include 38 AD subjects, 37 MCI subjects, 65 normal control subjects, and 3 unclassified subjects.

**Table 1:** Demographic information.

SUVR	<1.3	1.3∼1.5	1.5∼2.0	>2.0	PiB-	PiB+
Number of subjects Total (Female)	36 (21)	32 (14)	17 (11)	58 (26)	68 (35)	75 (37)
Age mean (STD)	74.2 (6.5)	73.2 (7.2)	70.4 (12.3)	76.1 (8.6)	73.7 (6.8)	74.8 (9.8)
MMSE mean (STD)	28.2 (2.0)	27.7 (2.7)	25.8 (3.5)	25.4 (5.1)	27.9 (2.4)	25.5 (4.8)

We sorted all subjects according to their SUVRs and uniformly sampled a subset of 20 cases (with both MRI and PET images) from the sorted list as an atlas set. In this way, the atlas set was drawn from the full range of PiB SUVR (Table 2). The atlas set includes 5 AD subjects, 5 MCI subjects, and 10 normal control subjects.

**Table 2:** SUVR of Atlases.

SUVR	<1.3	1.3∼1.6	1.6∼2.0	>2.0	PiB-	PiB+
No. of Atlases	4	5	3	8	9	11

### Methods

**Figure 1:**
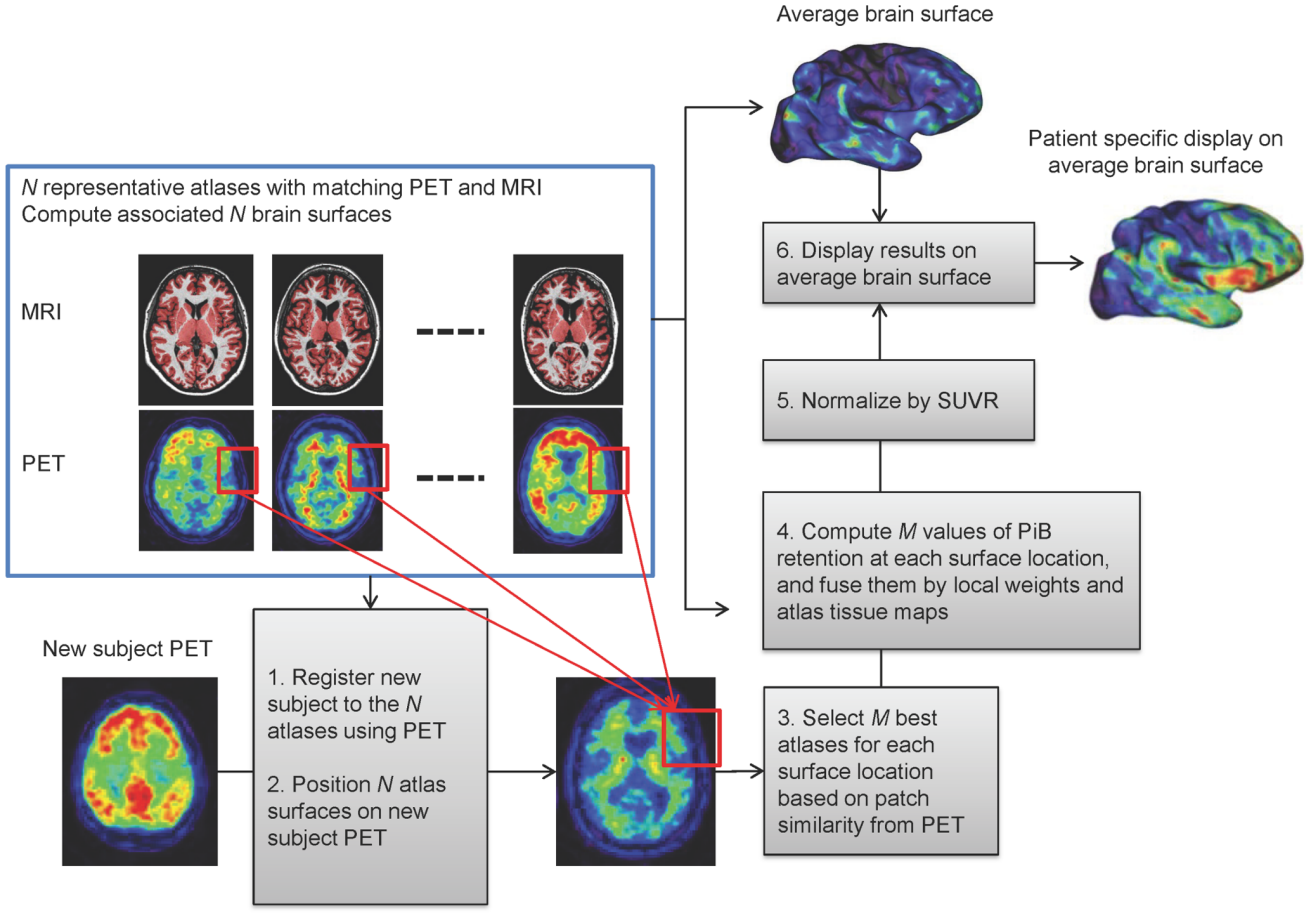
Overview of the proposed method.

An overview of the method is shown in Figure 1 and briefly described here.

For each new subject (without MRI),

- 1)Align priors: the PET image was aligned to each atlas PET image (Section “Affine registration of PET images”), which includes a GM tissue probability, and a cortical surface. The generation of these atlases is described in Section “Atlas generation”.
- 2)Locally and adaptively select optimal M atlases for each cortical location (Section “Local atlas selection”).
- 3)Generate consensus PiB estimate for each region using a Bayesian framework (Section “Surface-based estimation by multi-atlas fusion”).

The final output from this approach is a cortical surface (with atlas correspondence) with each vertex encoding the raw PiB retention estimation for that location.

This surface can then be SUVR normalized [24], [4] and used in visual reading (e.g. for clinical diagnosis) or used in population studies.

#### Atlas generation

The atlas set comprised twenty subjects with both PET and MR images (the atlases). All the atlases images were spatially normalized in two steps i) rigid registration [22] between the PET and MR images of the same subject; and ii) a non-linear registration [28] between the MR images of different subjects to the MNI space [19]. The aligned MRI images were further segmented into GM, WM and CSF tissue maps by the implementation of the segmentation algorithm in [2] with topological constraints that force the GM to be a continuous layer covering the WM [29]. GM segmentation was also topologically corrected in deep sulci [29]. A surface between GM and WM was computed using an expectation maximization scheme [2]. The cortical correspondence among the population of GM-WM atlas surfaces was computed using a multi-scale non–rigid surface registration EM-ICP algorithm [6]. An arbitrary surface was selected as the reference, with all other atlas surfaces registered and resampled. Each surface mesh consisted of 81922 vertices.

#### Affine registration of PET images

Each subject PET image was aligned to the 20 PET atlases using an affine registration based on block matching of feature points in PET images [22]. This method iteratively pairs image blocks, computes the corresponding transformation via maximizing normalized cross correlation, and then transforms the feature points accordingly until convergence. After registration, the surface and the probability maps were propagated into subject space.

#### Local atlas selection

To account for differences between the subject and the 20 atlases, only 10 atlases were selected using local matching. For each surface vertex, PET image similarity between the subject and an atlas was computed in a 30×30×30 (voxels) neighborhood by normalized mutual information (NMI) [31]. The ten most similar atlas PET images were selected to generate the final estimation at each vertex via a Bayesian fusion scheme explained in the next section.

#### Surface-based estimation by multi-atlas fusion

Given a PET image 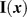, where 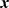 denotes an image voxel, we aimed at measuring the mean PiB retention in grey matter along the normal direction of the transformed atlas surface S^T^. That equals to estimate the expectation 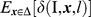, where 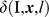 is an indicator function:
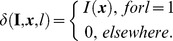



The symbol **Δ** denotes the intersection of the line along the normal direction of a surface vertex ***v*** and the PET image **I**. The symbol 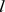 is the tissue label, representing GM, WM and CSF with the values of 1, 2 and 3, respectively. Taking discrete probability, we have 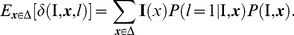
(1)


Assuming that ***x*** is evenly sampled from Δ, the probability 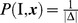, where |Δ| is the length of Δ. The posterior label probability 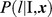 was estimated from the transformed atlases 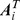 (*i* = 1 · · · *n*, with *n* the number of atlases selected in Section “Local atlas selection” by marginalizing the joint probability 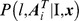: 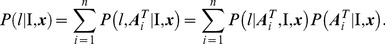
(2)



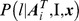
 represents the probability for the voxel ***x*** to be GM in the transformed atlas 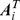, which was obtained in our case from the transformed atlas probability maps. The probability 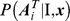 measures the probability of the voxel ***x*** to be well aligned between the test image **I** and the transformed atlas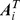. In our approach, 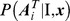 was set proportional to the reciprocal of the metric of normalized mutual information estimated locally within the neighborhood 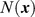 of ***x***. That is, 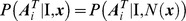. As mentioned in Section “Local atlas selection”, the size of 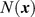 should be large to avoid fitting noise but small enough to fit local information. In our approach, 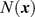 was set to be 30×30×30 (voxels), covering all the voxels along the line Δ. Therefore, 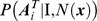 was constant with respect to the variable ***x*** (***x*** ∈ Δ).

Combining (1) and (2), we have 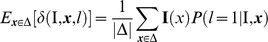





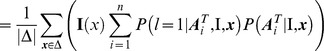





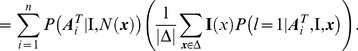
(3)


Equation (3) shows the additive property for the estimation of the mean PiB retention at each surface vertex: the estimation from multiple atlases can be attained by independent estimation from each single atlas and then linearly combined in a weighted scheme. The combination weights 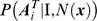 reflect the alignment between the test image and the transformed atlas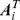. As the alignment is assessed by local metric, such a combination is nonlinear for the whole surface. This additive property is important for our approach where the atlas set needs to be dynamically determined. It makes the switch of selected atlases easy by confining the changes to the affected atlases only, thus being computationally efficient.

#### SUVR Normalization

PiB retention was normalized using SUVR [24], [18], [12]. The intensity values 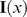 in the original PET image were homogeneously scaled by a parameter k. As NMI matches structures instead of intensity values, 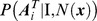 is invariant to k, and so is the GM probability 
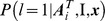
. Thus, we have 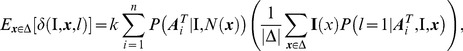
(4)


where k is determined by SUVR. Eqn. (4) shows that when SUVR changes, our estimation (3) can be simply scaled by the parameter k. Methods to perform PET-only SUVR normalization were proposed in [24], [4], which can segment the cerebellum in PET images without MRI.

#### Surface Visualization

AD patients usually have higher Aβ burdens than the healthy population. Such difference can be converted into z-scores based on the mean and standard deviation of the Aβ burden in PiB- healthy subjects. To facilitate clinical inspection, surface based z-score maps were generated, so that the unusual Aβ accumulation could be immediately identified and localized. Once the z-score maps were computed for each subject, the left and right hemispheres were assembled and visualized from six perspectives to generate a clinician friendly report.

### Validation

**Figure 2:**
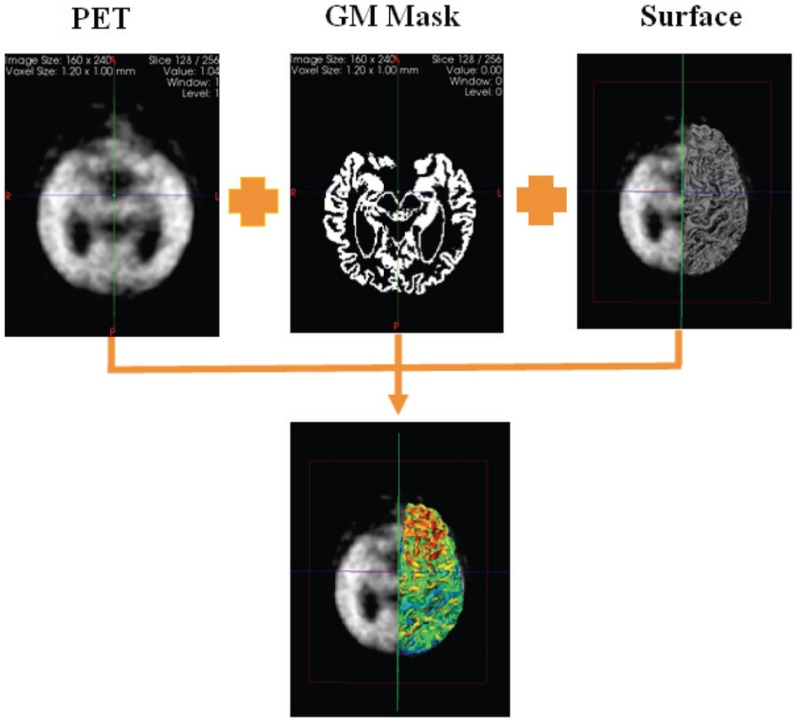
Illustration of the MRI-dependent method. The PiB retention is measured in the PET image within its grey matter mask obtained from MRI tissue segmentation, and averaged along the normal direction of the GM-WM interface (overlaid on the PET image) extracted from the subject's MRI. The mean PiB value for each surface vertex is mapped onto the surface for visualization.

The proposed method was compared to the MRI-dependent method, which is illustrated in Figure 2. The MRI image of each subject was segmented into three tissues of GM, WM and CSF (same as in the proposed method). The surface of MRI GM/WM interface was extracted and registered with the atlas surfaces using the multi-resolution EM-ICP method as described in Section “Atlas generation”. The subject surface (used in MRI-dependent method) and the atlas surface (used in the proposed PET-only method) share the same number of corresponding vertices allowing direct comparison. PiB retention was measured along the normal directions of the subject surface and averaged within the MRI GM mask. The obtained mean PiB retention was mapped onto the subject surface and visualized as in the proposed method.

The difference in PiB estimation between the MRI-dependent and the proposed methods was measured by absolute values (abs) or ratios (%) averaged over the total subjects. Such measurements were conducted at both the vertex and the Region of Interest (ROI) levels. In other words, at the vertex level we took each vertex as the comparison unit, while at the ROI level we took each ROI as the comparison unit. The ROIs were those from [35], which were mapped onto the MRI.

More specifically, two errors were computed:
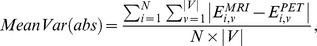





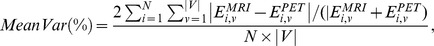
where 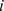 is the index of a subject, and 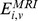 and 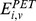 are the corresponding estimations at the 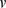-th vertex. The value of 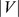 is set to the total number of vertices within the scope of the comparison that could be either the whole surface or a specific ROI.

Similarly, at the ROI level, two errors were computed:
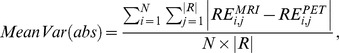


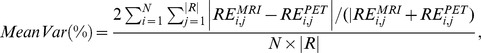
where 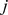 is the index of an ROI, and 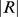 is the total number of the involved ROIs. If the comparison is conducted for a specific ROI, we set 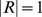. Here 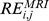 and 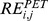 represent the averaged estimations for the 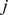-th ROI, which were computed as 
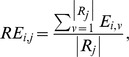



where 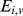 is defined as before, the estimation at the 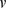-th vertex of the 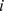-th subject. 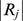 is the size (the number of vertices) of the 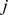-th ROI.

Z-score estimation was validated at each surface vertex by comparing the estimations from the MRI-dependent and the PET-only methods. The z-score of an estimation at the 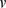-th vertex of the 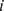-th subject was computed by 
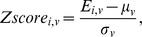
where 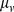 is the mean and 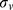 is the standard deviation of the estimations at the 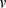-th vertex from the PiB- normal subjects. Z-score maps were used to detect the deviation of a subject from an asymptomatic control group using the MRI-dependent estimation of PiB retention. To calculate the z-scores of a new subject, 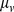 and 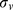 were computed over all 67 PiB- normal subjects in our data set (removing the new subject if it were PiB-). That is, for the 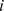-th PiB- normal subject, we assumed that the ground truth were known for all except the 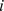-th PiB- normal subjects, and computed the 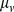 and 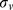 over these subjects. Although slightly different for different PiB- normal subjects, the same 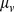 and 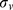 were used when computing 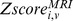 and 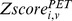 for a given subject 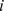, cancelling its influence when computing 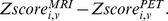 The mean and the standard deviation (STD) of the z-score differences between the two methods were calculated as 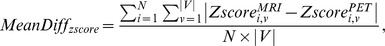


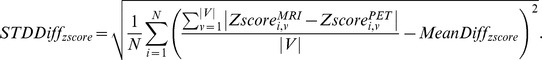



As defined above, 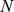 is the number of total subjects and 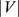 the number of total surface vertices.

## Results

In the following sections, results were presented by using the MRI-dependent assessment as the ground truth. We demonstrated the accuracy of the PET-only method for both PiB retention estimation and z-score map estimation. Our proposed method was also compared to a naive single-atlas based PET-only method similar to that in [16]. In order to evaluate the performance of the proposed Aβ estimation method, the results presented used the same MR-based SUVR normalization for both the MRI-dependent and the PET-only methods.

### Comparison of PiB Retention Estimation

#### Visual inspection

**Figure 3:**
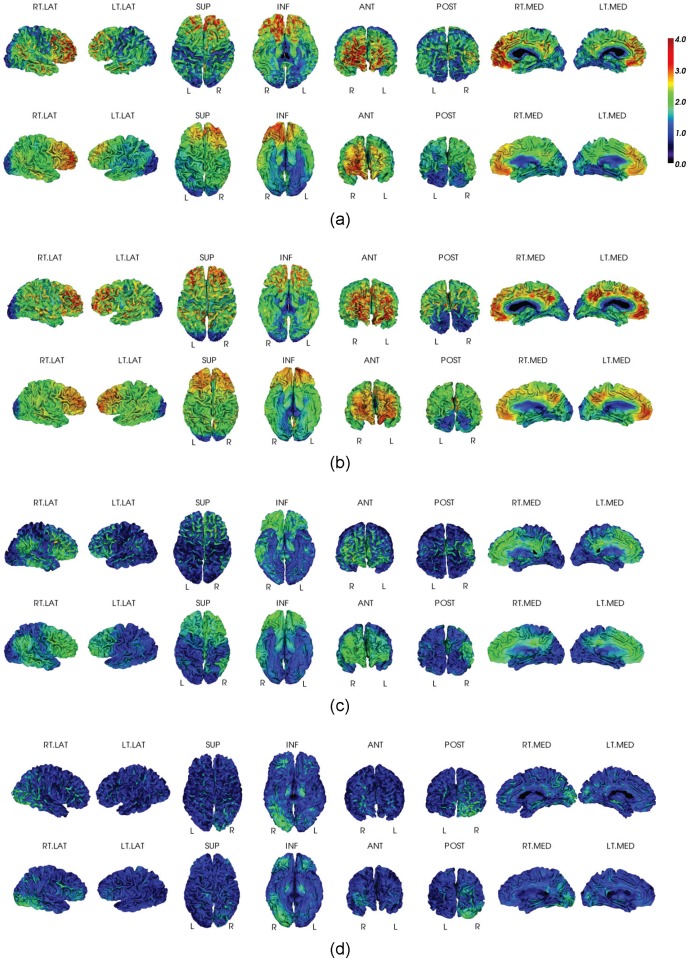
Visual Inspection for PiB measurements. Surface-based PiB measurements from the MRI-dependent method (the top row) and the proposed method (the bottom row) for four examples: (a) AD, (b) PiB+ NC, (c) PiB+ NC, and (d) PiB- NC.

Figure 3 shows four representative examples displaying the MRI-dependent (top row) and the PET-only (bottom row) methods: one PiB+ AD patient, one PiB+ NC (normal control) with high PiB retention, one PiB+ NC with low PiB retention, and one PiB- NC. The two methods present visually similar results for each subject.

#### Quantitative comparison

Quantitative analysis results are summarized in Table 3 and Table 4.

**Table 3:** Comparison between MRI-dependent and PET-only methods for PiB+ group (averaging over 123 subjects that are not included in the atlas set).

	All	Lateral Frontal Cortex	Occipital Cortex	Lateral Temporal Cortex	Parietal Cortex	Posterior Cingulate	Putamen
Mean MRI	1.87±0.27	1.87±0.28	1.86±0.27	1.87±0.27	1.86±0.27	1.88±0.28	1.83±0.26
Mean PiB	1.84±0.25	1.85±0.25	1.83±0.25	1.84±0.25	1.84±0.25	1.85±0.25	1.81±0.24
**ROI**							
Mean VAR (abs)	0.04±0.03	0.04±0.03	0.04±0.03	0.04±0.03	0.04±0.03	0.05±0.04	0.04±0.03
Mean VAR (%)	**2.15**±**1.5**	**2.26**±**1.6**	**2.13**±**1.5**	**2.08**±**1.4**	**2.11**±**1.5**	**2.42**±**1.7**	**2.04**±**1.4**
*Cited Mean VAR (%)	-	3.9	3.7	3.3	3.7	4.9	5.1
**Vertex**							
Mean VAR(abs)	0.23±0.04	0.23±0.04	0.23±0.04	0.22±0.04	0.23±0.04	0.23±0.04	0.23±0.04
Mean VAR (%)	13.4±1.2	13.4±1.2	13.3±1.2	13.1±1.2	13.4±1.2	13.5±1.5	13.6±1.6
Pearson Corr (R)	0.74±0.06	0.73±0.06	0.74±0.06	0.75±0.06	0.74±0.06	0.74±0.07	0.75±0.07
ICC	**0.94**	**0.94**	**0.94**	**0.94**	**0.94**	**0.94**	**0.94**
*Cited ICC	-	0.95	0.94	0.97	0.94	0.88	0.89

**Table 4:** Comparison between MRI-dependent and PET-only methods for PiB- group (averaging over 123 subjects that are not included in the atlas set).

	All	Lateral Frontal Cortex	Occipital Cortex	Lateral Temporal Cortex	Parietal Cortex	Posterior Cingulate	Putamen
Mean MRI	1.15±0.08	1.15±0.08	1.16±0.08	1.15±0.08	1.16±0.08	1.16±0.09	1.15±0.09
Mean PiB	1.19±0.08	1.19±0.08	1.19±0.08	1.19±0.08	1.19±0.08	1.19±0.08	1.19±0.08
**ROI**							
Mean VAR (abs)	0.04±0.03	0.04±0.03	0.04±0.03	0.04±0.03	0.04±0.03	0.04±0.03	0.05±0.03
Mean VAR (%)	**3.60**±**2.3**	**3.63**±**2.4**	**3.42**±**2.1**	**3.48**±**2.2**	**3.46**±**2.4**	**3.22**±**2.3**	**3.81**±**2.4**
*Cite Mean VAR (%)	-	2.7	3.2	2.5	2.0	0.9	4.1
**Vertex**							
Mean VAR(abs)	0.19±0.02	0.19±0.02	0.19±0.02	0.18±0.02	0.19±0.02	0.19±0.02	0.19±0.02
Mean VAR(%)	16.3±1.7	16.3±1.2	16.2±1.6	16.1±1.6	16.5±1.7	16.3±1.7	16.5±1.8
Pearson Corr (R)	0.42±0.1	0.42±0.1	0.43±0.1	0.42±0.1	0.41±0.1	0.43±0.1	0.41±0.1
ICC	**0.72**	**0.72**	**0.72**	**0.72**	**0.71**	**0.76**	**0.68**
*Cited ICC	-	0.73	0.75	0.69	0.59	0.75	0.66

In Table 3 and Table 4, “Mean MRI” and “Mean PiB” are the mean ROI PiB retention estimated by the MRI-dependent and PET-only methods respectively. The estimation differences (mean VAR) are measured at both ROI and vertex level, as shown in the tables. The Pearson correlation and the intra-class correlation (ICC) are computed by correlating the estimations from the two methods at each vertex, respectively.

**Figure 4:**
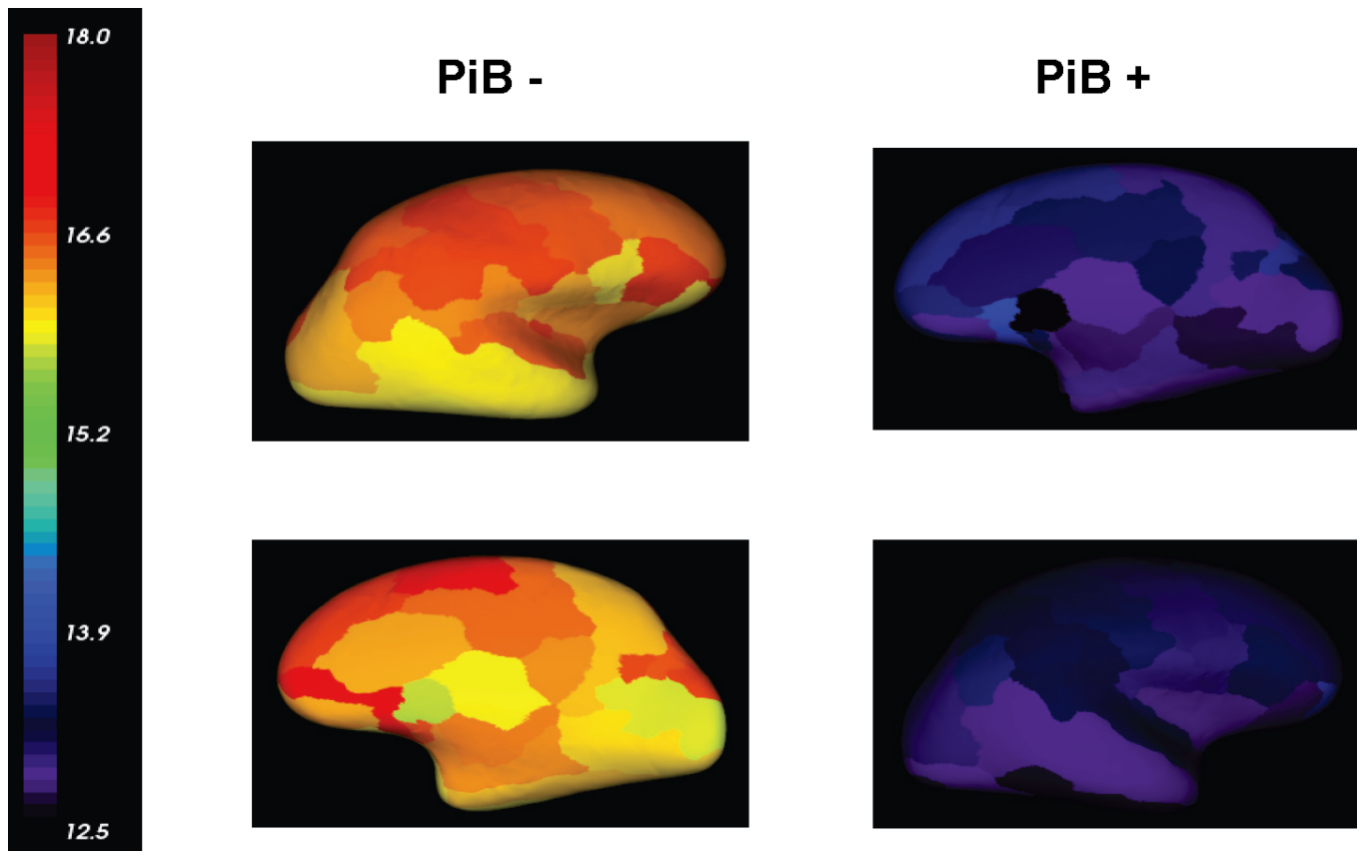
Vertex-based mean estimation errors (ratio) in each AAL ROI. The errors are visualized on an inflated template brain surface for both PiB+ and PiB- groups. There are higher estimation error ratios for PiB- group than for PiB+ group, due to the minimal amount of retention and the reduced dynamic range of PiB- group. The mean absolute estimation error (vertex-based) is 0.19±0.03 for PiB- group and 0.23±0.04 for PiB+ group as reported in Table 3 and Table 4.

The PiB+ group had lower VAR ratio (2.15±1.5% for ROI, 13.4±1.2% for vertex) than the PiB− group (3.6±2.3% for ROI, 16.6±1.5% for vertex), as well as attaining higher Pearson correlation/ICC (0.74/0.94) than the PiB- group (0.42/0.72). This difference was expected, because the PiB− group has minimal PiB retention, a reduced dynamic range and is therefore more susceptible to noise. The accuracy in the PiB− group allowed identifying similar patterns to the MRI-dependent method (Figure 4 (d)), with an absolute VAR of 0.04±0.03 for ROI, and 0.19±0.02 for vertex. We also found that our estimation errors for both groups were close to the reported reproducible errors of PiB quantification using 30 min imaging [1] (cited in Table 3 and Table 4).

**Figure 5:**
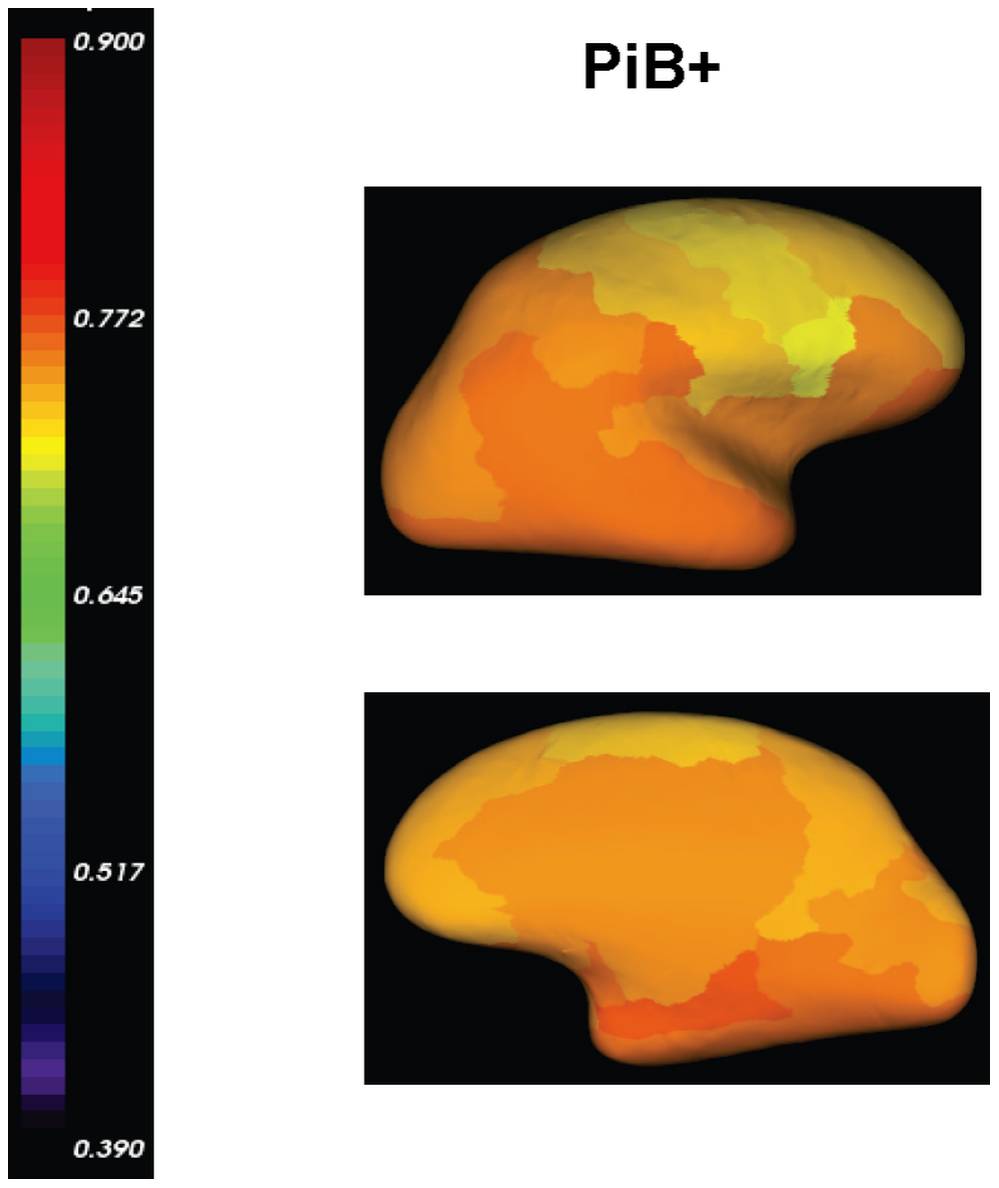
Mean correlations between PET-only and MRI-dependent methods over AAL ROIs. The correlations are visualized on inflated template brain surface for both PiB+ and PiB- groups.

In addition to the selected ROIs listed in Table 3 and 4, the vertex-based mean estimation error (in ratio) and the mean correlation at each AAL ROI [35] are also presented in Figure 4 and Figure 5, respectively. The results were visualized on an inflated template surface for easier interpretation. The PiB+ group had lower errors and higher correlations consistently in all AAL ROIs.

**Figure 6:**
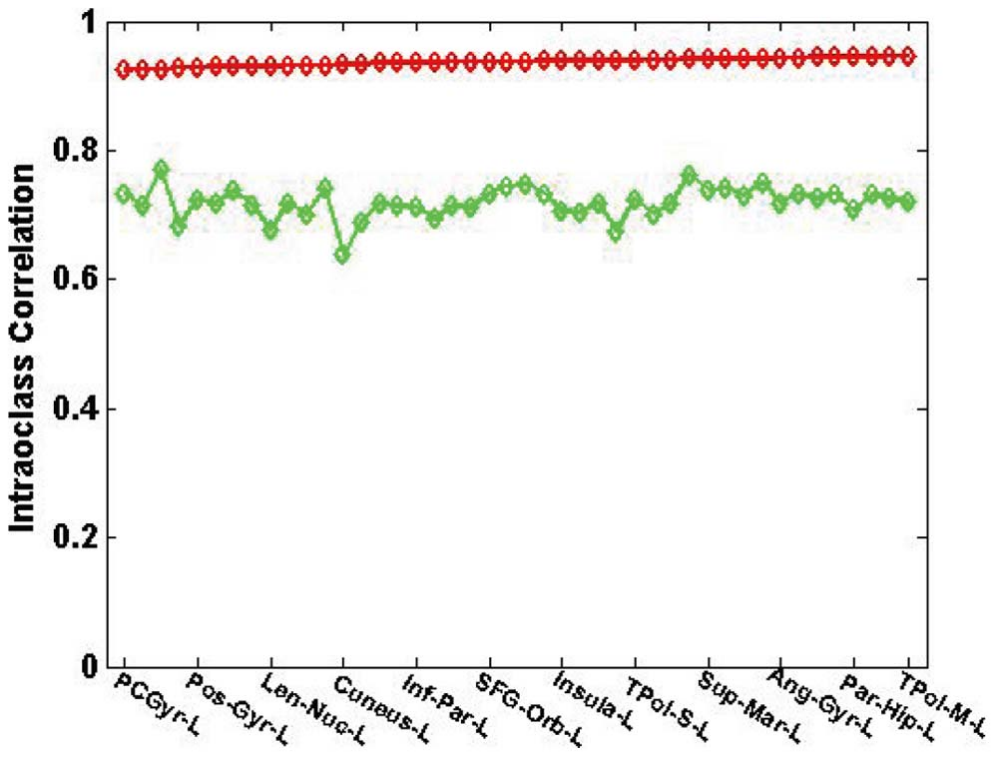
Intra-class correlation over AAL ROIs between PET-only and the MRI-dependent methods. Red line is for the PiB+ group and green line is for the PiB- group. To improve clarity, not all ROI names are shown in the graph.

To remove the ambiguity of invariance to linear transformations existing in Pearson correlation, the intra-class correlation (ICC) was also computed by correlating all the mean PiB estimations (averaged over all subjects in the PiB+ and PiB- groups, respectively) at each surface vertex between the two methods. The ICCs for all AAL ROIs are presented in Figure 6. The corresponding full and short ROI names are given in File S1. The ICC is homogeneous and greater than 0.9 for ROIs in the PiB+ group, while still reaching 0.7 in average for the PiB- group despite the low signal of this group.

### Comparison of Z-score Estimation

The estimated z-scores at each surface vertex were compared between the MRI-dependent and the PET-only methods. The z-score difference and the Pearson correlation were computed and presented in Table 5. Similarly to the PiB retention estimation, the z-scores for PiB+ group from the PET-only method agreed well with that from the MRI-dependent method with a mean difference of 0.94 per vertex and a standard deviation of 0.2, and a Pearson correlation R = 0.81. The PiB- group was found to have a mean z-score difference of 0.71 per vertex and a standard deviation of 0.1, and a Pearson correlation R = 0.4 between the PET-only and the MRI-dependent estimations.

**Table 5:** Comparison of Z-score Estimation between the PET-only and the MRI-dependent Methods (averaging over 123 subjects that are not in the atlas set).

	Z-score Difference: (per vertex) ±	Pearson Correlation (R)
PiB+ Group	0.94±0.2	0.81
PiB- Group	0.71±0.1	0.40

**Figure 7:**
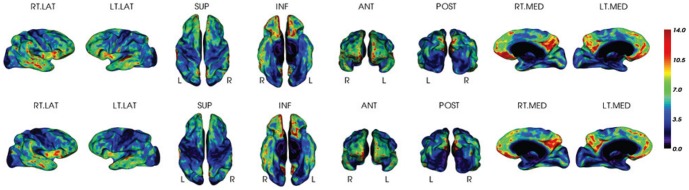
Mean Z-score for PiB+ AD group. The Z-scores are estimated by the MRI-dependent (top row) and the PET-only (bottom row) methods, respectively.

**Figure 8:**
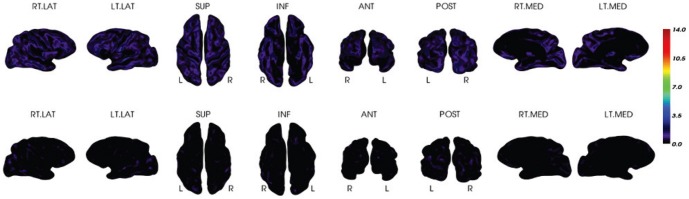
Mean Z-score for PiB- NC group. The Z-scores are estimated by the MRI-dependent (top row) and the PET-only (bottom row) methods, respectively.

**Figure 9:**
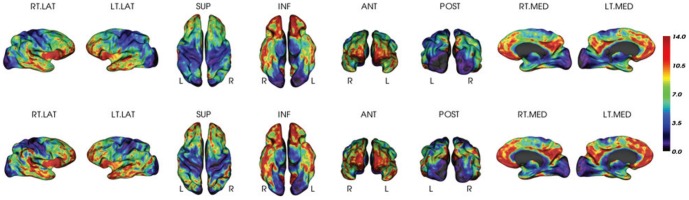
Z-score for an individual PiB+ subject. It is estimated by the MRI-dependent (top row) and the PET-only (bottom row) methods, respectively.

**Figure 10:**
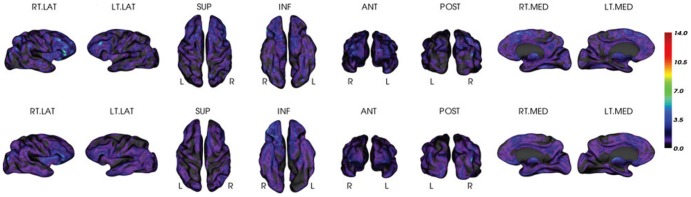
Z-score for an individual PiB- subject. It is estimated by the MRI-dependent (top row) and the PET-only (bottom row) methods, respectively.

For a visual comparison, Figure 7 and Figure 8 display the mean z-scores averaged over the PiB+ AD group and the PiB- NC group, respectively. It can be seen that the z-scores for both PiB+ and PiB- groups are similar between the two methods. Two individual examples are given in Figure 9 and Figure 10, where similar levels and pattern of PiB retention can be observed.

### Comparison of Multiple Atlas vs. Single Atlas

**Figure 11:**
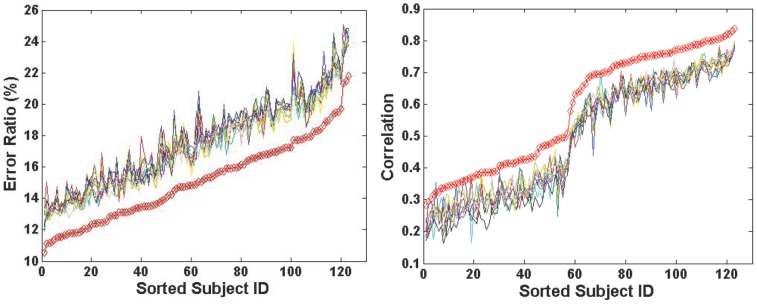
Comparison between the multiple-atlas and ten single-atlas approaches subject by subject (left: error ratio; right: correlation). The red line corresponds to the proposed multiple-atlas approach, while the rest ten lines correspond to the ten single-atlas approaches in comparison, respectively. To improve clarity, the subjects' IDs are sorted according to the increase of error ratios and correlation, respectively.

To demonstrate the advantage of using multiple atlases, the average estimation error ratios for each subject and the correlations with the MRI-dependent method were compared between using any randomly selected single-atlas approaches and the proposed multiple-atlas approach. The results were plotted in Figure 11. The red line corresponds to the result from the multiple-atlas approach, and the ten other lines correspond to ten single-atlas approaches. The estimation from the multiple-atlas approach consistently exhibits significantly lower average estimation errors and higher correlations over the 123 test subjects compared to the best results obtained using any single atlas.

**Figure 12:**
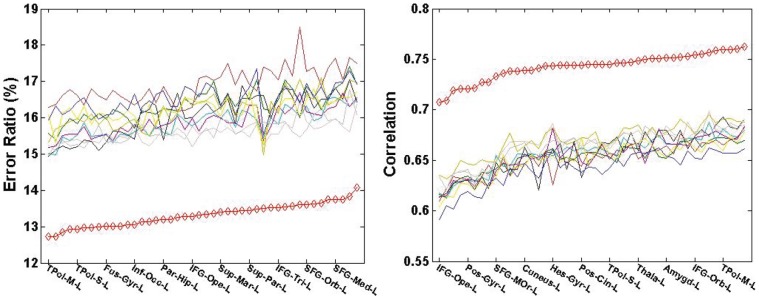
Comparison of the multiple-atlas and ten single-atlas approaches over AAL ROIs within PiB+ group (left: error ratio; right: correlation). The red line corresponds to the proposed multiple-atlas approach, while the rest ten lines correspond to the ten single-atlas approaches in comparison, respectively. To improve clarity, not all ROI names are shown in the graph.

**Figure 13:**
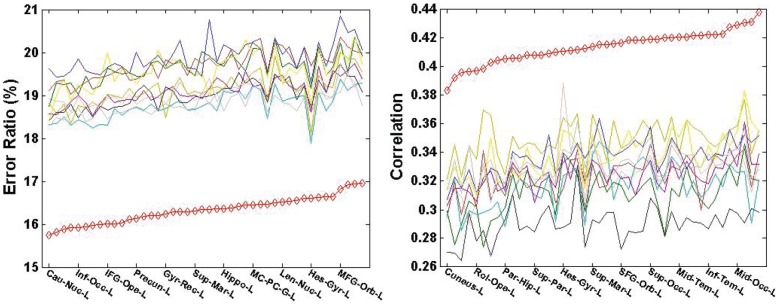
Comparison of the multiple-atlas and ten single-atlas approaches over AAL ROIs within PiB- group (left: error ratio; right: correlation). The red line corresponds to the proposed multiple-atlas approach, while the rest ten lines correspond to the ten single-atlas approaches in comparison, respectively. To improve clarity, not all ROI names are shown in the graph.

Moreover, the estimation error ratios and the correlations are further broken into surface ROIs according to the AAL atlas to explore the local estimation performance. Within each ROI, the estimation error ratio and the correlation are averaged over all the subjects in PiB+ and PiB- groups, respectively. The results are summarized in Figure 12 and Figure 13. The red line corresponds to the result from the multiple-atlas approach, and the other ten lines correspond to the ten single-atlas approaches. The results reveal a pronounced advantage of the proposed multi-atlas approach (when averaged over the groups) over any single-atlas approach in all the AAL ROIs for both PiB+ and PiB- groups.

## Discussion

We developed a surface-based method to quantify and visualize PiB retention within cortical GM without the need of MR images. Our proposed PET-only method showed good correlation with the more traditional MRI-dependent method. The variation between the two methods was similar to published test-retest result of PiB quantification [1]. As PiB retention within GM may indicate Aβ deposition associated with Alzheimer's disease, our proposed method could be used as a clinical tool to help physicians to easily determine the Aβ burden of patients, improving diagnostic confidence. Future studies will be performed to investigate and validate the clinical utility of the proposed method.

Our method compared very favorably against the single-atlas based PET-only method. Although no quantitative analysis about the precision was reported in [16], we found that the performance of single-atlas based method was highly dependent on the atlas selection due to the different anatomy between the single atlas and the subject. For example, for some given subject, the estimation error ratio (per vertex) varied from 15% to 25% when different individual atlases were used from the pool of the 20 atlases.

Previous work in MRI has found that multiple atlases allowed averaging registration errors and increasing robustness via some consensus method [3]. However, the optimal way of selecting atlases and combining them is application-dependent. In our case, PiB retention in the population is quite heterogeneous (globally) and patchy (locally) both in terms of shape and appearance. In order to address this variation, we adopted several strategies to benefit from the use of multiple atlases. Firstly, we set up a pool of atlases that covered the whole spectrum of the disease. As changes in PiB retention are believed to reflect Aβ progressive accumulation, the atlases were therefore chosen to match the full range of PiB cases. Secondly, a subset of 10 atlases was selected from the atlas pool according to the closest “local” matching of appearance. Using a subject-specific subset of atlases reduced the negative influence of dissimilar atlases to that subject. We also found that “local” matching had better capacity to handle the inter-subject variation than “global” matching. In our case, using global matching for multiple atlases could not outperform a good single atlas based approach. Thirdly, the estimations from each atlas were “locally” weighted in a Bayesian fusion framework. This framework also involved a probabilistic tissue map that considered the variation within the training population. These strategies allowed locally adaptive estimation of the PiB retention to be made, which reduced errors due to mismatches in the distribution of the plaques and GM shapes. This is important and allowed our method to successfully handle more unusual or early stage (asymmetries, focal retention) subjects. The parameters that we used (10 most similar out of 20 atlases) were found to provide the best results. Whether using different number for different cohorts would be more optimal remains to be investigated.

The performances of the proposed method were excellent for the PiB+ group and better than for the PiB- group. This is expected since PiB- subjects have low PiB retention in the GM, and therefore a very low SNR. This low SNR decreases the performance when using percentage, although it still provides very robust results when measured in absolute signal variation. The visualization of PiB- subjects could still provide insight into the pattern of retention over the brain surface, with very low average difference compared to MR-dependent method, and an absolute estimation difference (per vertex) of 0.19±0.03. The error of 0.19 compares to the variability of PiB which has been shown to be 4-7% [36], [18], [34]. The good performance of this new method warrants further investigation to establish the benefit of using such a visualization tool compared to the traditional 3D visualization used in clinical practice.

The work in [1] evaluated the reproducibility of ^11^C PiB quantification at both the regional and the voxel levels when using only 30 min (60 to 90 min after tracer injection) of imaging data. Although their purpose was different from ours, the test-retest results indicated a possible range of variability for ^11^C PiB quantification. Being close to that range showed that the performance of our proposed PET-only method is reasonably consistent with that of the traditional MR-dependent method. In Table 3 and 4, our ROI-level VAR (%) was compared to that of the ROI measurements in [1]. The voxel-level VAR in [1] was not referenced because our vertex-level measurement was averaged over voxels along the normal direction of the GM/WM surface and therefore was incomparable to the voxel-level measurement in [1]. Meanwhile, our vertex-level ICC resembled more closely to the ROI-level ICC in [1] as cited. The voxel-level ICCs in [1] were much worse than their ROI-level ICCs, and hence worse than that of our method, too.

The amyloid assessment is affected by the partial volume effect and the resolution of the PET images, in both the traditional MRI-dependent and our method. Partial volume effect (PVE) appears when the size of the PET point spread function is greater than the image resolution, resulting in signal spill over from one tissue with high radiotracer concentration to one with low radiotracer concentration. Low resolution of PET images may lead to relatively large estimation errors in thin cortical structures, especially in the presence of PVE. This situation could be improved by using a partial volume correction method based on MRI [25], [23]. However, to our knowledge those PVE correction methods are seldom used in clinical workflow. As for higher resolution PET images, as long as the traditional MRI-dependent method could benefit from them, our PET-only method could also be improved simultaneously because we utilized the atlases' MRI and PET to estimate the PiB PET of the new subjects.

There are several limitations to the current study that should be acknowledged. The first is that the method is validated using the imaging protocols and scanners in the AIBL study. Further study is required to ensure that the method generalizes to other populations, studies and imaging protocols, especially if the PET reconstruction is significantly changed. It is also acknowledged that this paper utilizes ^11^C PiB PET, which, despite its wide use in dementia research to assess Aβ amyloid burden *in vivo*, is becoming less relevant with the recent development of various ^18^F-labeled radiotracers that have longer radioactive decay half-life and thus may be less restrictive in clinic use [37]. Although we expect similar performance, future work is required to validate and report the performance of our method using these tracers. Meanwhile, the clinical utility of the surface visualization should also be consolidated.

## Conclusion

In the present study, we propose an approach to estimate Aβ burden utilizing only ^11^C PiB PET images. This reduces the necessity of acquiring MRI images to perform accurate quantification using conventional methods. This is achieved by taking advantages of an automatic selection of subject-specific optimal atlases and an effective multiple atlas fusion scheme. Our MR-less method applied to a large cohort of images from AIBL was validated against an MR based method, demonstrating the accuracy and robustness of the PiB retention estimation.

## Supporting Information

File S1 **AAL ROI names (short vs full) used in the paper.**
(DOCX)

## References

[1] Aalto S, Scheinin MN, Kemppainen MN, Någren K, Kailajärvi M, et al (2009) Reproducibility of automated simplified voxel-based analysis of PET amyloid ligand [11C]PIB uptake using 30-min scanning data. Eur J Nucl Med Mol Imaging 36: 1651–1660. 19495749 10.1007/s00259-009-1174-1

[2] Acosta O, Fripp J, Doré V, Bourgeat P, Favreau JM, et al (2012) Cortical surface mapping using topology correction, partial flattening and 3D shape context-based non-rigid registration for use in quantifying atrophy in Alzheimer's disease. Journal of Neuroscience Methods 205: 96–109. 22226742 10.1016/j.jneumeth.2011.12.011

[3] Artaechevarria X, Munoz-Barrutia A, Ortiz-de-Solorzano C (2009) Combination strategies in multi-atlas image segmentation: application to brain MR data. IEEE Trans Med Imaging 28 (8): 1266–1277. 10.1109/TMI.2009.2014372 19228554

[4] Bourgeat P, Raniga P, Dore V, Zhou L, Macaulay SL, et al.. (2012) Manifold Driven MR-less PiB SUVR Normalisation. In MICCAI 2012 Workshop on Novel Imaging Biomarkers for Alzheimer's Disease and Related Disorders (NIBAD'12)

[5] Braak H, Braak E (1991) Neuropathological stageing of Alzheimer-related changes. Acta Neuropathol. (Berl) 82 (4): 239–259. 10.1007/BF00308809 1759558

[6] Doré V, Fripp J, Bourgeat P, Shen K, Salvado O (2011) Surface-base Approach using a Multi-Scale EM-ICP Registration for Statistical Population Analysis. In Proceedings of the Digital Image Computing Techniques and Applications (DICTA) 2011: 13–18.

[7] Ellis KA, Bush AI, Darby D, De Fazio D, Foster J, et al (2009) The Australian Imaging, Biomarkers and Lifestyle (AIBL) study of aging: methodology and baseline characteristics of 1112 individuals recruited for a longitudinal study of Alzheimer's disease. Int Psychogeriatr 21: 672–687. 19470201 10.1017/S1041610209009405

[8] Fripp J, Bourgeat P, Acosta O, Raniga P, Modat M, et al (2008) Appearance modeling of 11C PiB PET images: characterizing amyloid deposition in Alzheimer's disease, mild cognitive impairment and healthy aging. Neuroimage 43 (3): 430–439. 10.1016/j.neuroimage.2008.07.053 18789389

[9] Fodero-Tavoletti T, Rowe C, McLean A, Leone L, Li X, et al (2009) Characterization of PiB binding to white matter in Alzheimer disease and other dementias. J Nucl Med 50(2): 198–204. 19164220 10.2967/jnumed.108.057984

[10] Hammers A, Allom R, Koepp J, Free L, Myers R, et al (2003) Three-dimensional maximum probability atlas of the human brain, with particular reference to the temporal lobe. Hum. Brain Mapp 19(4): 224–247. 12874777 10.1002/hbm.10123 PMC6871794

[11] Jack CR Jr, Lowe VJ, Senjem ML, Weigand SD, Kemp BJ, et al (2008) 11C PiB and structural MRI provide complementary information in imaging of Alzheimer's disease and amnestic mild cognitive impairment. Brain 131: 665–680. 18263627 10.1093/brain/awm336 PMC2730157

[12] Joachim C, Morris J, Selkoe D (1989) Diffuse senile plaques occur commonly in the cerebellum in Alzheimer's disease. American J. Pathol. 135 (2): 309–319. PMC1879919 2675616

[13] Kemppainen M, Aalto S, Wilson IA, Nagren K, Helin S, et al (2006) Voxel-based analysis of PET amyloid ligand [11C]PiB uptake in Alzheimer disease. Neurology 67: 1534–1535. 10.1212/01.wnl.0000240117.55680.0a 16971697

[14] Kemppainen M, Aalto S, Wilson I, Nagren K, Helin S, et al (2007) PET amyloid ligand [11C] PiB uptake is increased in mild cognitive impairment. Neurology 68 (5): 1603–1606. 10.1212/01.wnl.0000260969.94695.56 17485647

[15] Klunk E, Engler H, Nordberg A, Wang Y, Blomqvist G, et al (2004) Imaging brain amyloid in Alzheimer's disease with Pittsburgh Compound-B. Ann. Neurol. 55 (3): 306–319. 14991808 10.1002/ana.20009

[16] Lilja, A., Thurfjell L., 2010. Tools for aiding in the diagnosis of neurodegenerative diseases. http://www.faqs.org/patents/app/20100080432

[17] Li Y, Rinne JO, Mosconi L, Pirraglia E, Rusinek H, et al (2008) Regional analysis of FDG and PIB-PET images in normal aging, mild cognitive impairment, and Alzheimer's disease. Eur. J. Nucl. Med. Mol. Imaging. 35 (12): 2169–2181. 18566819 10.1007/s00259-008-0833-y PMC2693402

[18] Lopresti BJ, Klunk WE, Mathis CA, Hoge JA, Ziolko SK, et al (2005) Simplified quantification of Pittsburgh Compound B amyloid imaging PET studies: a comparative analysis. J Nucl Med. 46 (12): 1959–1972. 16330558

[19] Mazziotta J, Toga A, Evans A, Fox P, Lancaster J, et al (2001) A probabilistic atlas and reference system for the human brain. Philos Trans R Soc Lond B Biol Sci 356: 1293–1322. 11545704 10.1098/rstb.2001.0915 PMC1088516

[20] Ng S, Villemagne L, Berlangieri S, Lee T, Cherk M, et al (2007) Visual assessment versus quantitative assessment of 11C-PiB PET and 18F-FDG PET for detection of Alzheimer's disease. J. Nucl. Med. 48 (4): 547–552. 17401090 10.2967/jnumed.106.037762

[21] Okello A, Koivunen J, Edison P, Archer A, Turkheimer E, et al (2009) Conversion of amyloid positive and negative MCI to AD over 3 years: an 11C-PIB PET study. Neurology 73(10): 754–760. 19587325 10.1212/WNL.0b013e3181b23564 PMC2830881

[22] Ourselin S, Roche A, Subsol G, Pennec X, Ayache N (2001) Reconstructing a 3D structure from serial histological sections. Image Vis. Comput. 19 (1): 25–31.

[23] Raniga P, Bourgeat P, Fripp J, Acosta O, Ourselin S, et al.. (2009) Alzheimer's disease detection using 11C-PiB with improved partial volume effect correction. In SPIE: Medical Imaging 2009.

[24] Raniga P, Bourgeat P, Villemagne V, O'Keefe G, Rowe C, et al (2007) PIB-PET Segmentation for Automatic SUVR Normalization Without MR Information. In Proceedings of IEEE International Symposium on Biomedical Imaging: From Nano to Macro (ISBI) 2007: 348–351.

[25] Rousset OG, Ma Y, Evans AC (1998) Correction for partial volume effects in PET: Principle and validation. Journal Nucl Med 39(5): 904–911. 9591599

[26] Rowe CC, Ng S, Ackermann U, Gong J, Pike K, et al (2007) Imaging beta-amyloid burden in aging and dementia. Neurology 68 (20): 1718–1725. 10.1212/01.wnl.0000261919.22630.ea 17502554

[27] Rowe CC, Ellis KA, Rimajova M, Bourgeat P, Pike KE, et al (2010) Amyloid imaging results from the Australian Imaging, Biomarker and Lifestyle (AIBL) study of aging. Neurobiol. Aging 31: 1275. 20472326 10.1016/j.neurobiolaging.2010.04.007

[28] Rueckert D, Sonoda LI, Hayes C, Hill DLG, Leach MO, et al (1999) Nonrigid Registration Using Free-Form Deformations: Application to Breast MR Images. IEEE Trans. on Medical Imaging 18(8): 712–721. 10534053 10.1109/42.796284

[29] Rueda A, Acosta O, Couprie M, Bourgeat P, Fripp J, et al (2010) Topology-corrected segmentation and local intensity estimates for improved partial volume classification of brain cortex in MRI,” J. Neurosci. Methods 188: 305–315. 10.1016/j.jneumeth.2010.02.020 20193712

[30] Schott JM, Bartlett JW, Fox NC, Barnes J (2010) Increased brain atrophy rates in cognitively normal older adults with low cerebrospinal fluid Abeta1-42. Ann Neurol 68: 825–834. 21181717 10.1002/ana.22315

[31] Studholme C, Hill D, Hawkes D (1999) An overlap invariant entropy measure of 3D Medical image alignment. Pattern Recogn. 32 (1): 71–86.

[32] Thal DR, Rüb U, Orantes M, Braak H (2002) Phases of Abeta-deposition in the human brain and its relevance for the development of AD. Neurology 58 (12): 1791–1800. 12084879 10.1212/wnl.58.12.1791

[33] Thal DR, Capetillo-Zarate E, Del Tredici K, Braak H (2006) The development of amyloid beta protein deposits in the aged brain. Sci. Aging Knowledge Environ. 2006 (6). 10.1126/sageke.2006.6.re1 16525193

[34] Tolboom N, Yaqub M, Boellaard R, Luurtsema G, Windhorst AD, et al (2009) Test-retest variability of quantitative ^11^C PIB studies in Alzheimer's disease. Eur J Nucl Med Mol Imaging 36(10): 1629–1638. 19384547 10.1007/s00259-009-1129-6 PMC2758198

[35] Tzourio-Mazoyer N, Landeau B, Papathanassiou D, Crivello F, Etard O, et al (2002) Automated Anatomical Labeling of Activations in SPM Using a Macroscopic Anatomical Parcellation of the MNI MRI Single-Subject Brain. NeuroImage 15: 273–289. 11771995 10.1006/nimg.2001.0978

[36] Villemagne VL, Pike KE, Chételat G, Ellis KA, Mulligan RS, et al (2011) Longitudinal Assessment of Aβ and Cognition in Aging and Alzheimer Disease. Ann Neurol 69(1): 181–192. 21280088 10.1002/ana.22248 PMC3045039

[37] Villemagne VL, Ong K, Mulligan RS, Holl G, Pejoska S, et al (2011) Amyloid imaging with 18F-florbetaben in Alzheimer disease and other dementias. J Nucl Med 52: 1210–1217. 21764791 10.2967/jnumed.111.089730

[38] Yasuno F, Hasnine H, Suhara T, Ichimiya T, Sudo Y, et al (2002) Template-based method for multiple volumes of interest of human brain PET images. Neuroimage 16 (3 Pt 1): 577–586. 10.1006/nimg.2002.1120 12169244

[39] Yotter RA, Doshi J, Clark V, Sojkova J, Zhou Y, et al (2013) Memory decline shows stronger associations with estimated spatial patterns of amyloid deposition progression than total amyloid burden. Neurobiology of Aging. 34 (12): 2835–2842. 23859610 10.1016/j.neurobiolaging.2013.05.030 PMC3893024

[40] Ziolko K, Weissfeld A, Klunk E, Mathis A, Hoge A, et al (2006) Evaluation of voxel-based methods for the statistical analysis of PiB PET amyloid imaging studies in Alzheimer's disease. Neuroimage 33 (1): 94–102. 16905334 10.1016/j.neuroimage.2006.05.063

