# Supplementary material for: MR-Less Surface-Based Amyloid Assessment Based on 11C PiB PET
Source: PLoS One. 2014 Jan 10;9(1):e84777. doi: 10.1371/journal.pone.0084777 (PMC3888418; doi:10.1371/journal.pone.0084777)
Supplement: File S1 — AAL ROI names (short vs full) used in the paper. (DOCX) [file pone.0084777.s001.docx]

1 Precentral_gyrus_Left

2 Precentral_gyrus_Right

3 Superior_Frontal_gyrus,dorsolateral_Left

4 Superior_Frontal_gyrus,dorsolateral_Right

5 Superior_frontal_gyrus,orbital_part_Left

6 Superior_frontal_gyrus,orbital_part_Right

7 Middle_Frontal_gyrus_Left

8 Middle_Frontal_gyrus_Right

9 Middle_frontal_gyrus,orbital_part_Left

10 Middle_frontal_gyrus,orbital_part_Right

11 Inferior_frontal_gyrus,opercular_part_Left

12 Inferior_frontal_gyrus,opercular_part_Right

13 Inferior_frontal_gyrus,triangular_parti_Left

14 Inferior_frontal_gyrus,triangular_part_Right

15 Inferior_frontal_gyrus,orbital_part_Left

16 Inferior_frontal_gyrus,orbital_part_Right

17 Rolandic_Operculum_Left

18 Rolandic_Operculum_Right

19 Supplementary_motor_area_Left

20 Supplementary_motor_area_Right

21 Olfactory_cortex_Left

22 Olfactory_cortex_Right

23 Superior_frontal_gyrus,medial_Left

24 Superior_frontal_gyrus,medial_Right

25 Superior_frontal_gyrus,medial_orbital_Left

26 Superior_frontal_gyrus,medial_orbital_Right

27 Gyrus_rectus_Left

28 Gyrus_rectus_Right

29 Insula_Left

30 Insula_Right

AAL ROIs – Short Name in the paper

AAL ROIs – Full Name

1 PCGyr-L

2 PCGyr-R

3 SFG-Dor-L

4 SFG-Dor-R

5 SFG-Orb-L

6 SFG-Orb-R

7 MFGyr-L

8 MFGyr-R

9 MFG-Orb-L

10 MFG-Orb-R

11 IFG-Ope-L

12 IFG-Ope-R

13 IFG-Tri-L

14 IFG-Tri-R

15 IFG-Orb-L

16 IFG-Orb-R

17 Rol-Ope-L

18 Rol-Ope-R

19 Sup-Mot-L

20 Sup-Mot-R

21 Olf-Cor-L

22 Olf-Cor-R

23 SFG-Med-L

24 SFG-Med-R

25 SFG-MOr-L

26 SFG-MOr-R

27 Gyr-Rec-L

28 Gyr-Rec-R

29 Insula-L

30 Insula-R

31 AC-PC-G-L

32 AC-PC-G-R

33 MC-PC-G-L

34 MC-PC-G-R

35 Pos-Cin-L

36 Pos-Cin-R

37 Hippo-L

38 Hippo-R

39 Par-Hip-L

40 Par-Hip-R

41 Amygd-L

42 Amygd-R

43 Cal-Fis-L

44 Cal-Fis-R

45 Cuneus-L

46 Cuneus-R

47 Lin-Gyr-L

48 Lin-Gyr-R

49 Sup-Occ-L

50 Sup-Occ-R

51 Mid-Occ-L

52 Mid-Occ-R

53 Inf-Occ-L

54 Inf-Occ-R

55 Fus-Gyr-L

56 Fus-Gyr-R

57 Pos-Gyr-L

58 Pos-Gyr-R

59 Sup-Par-L

60 Sup-Par-R

31 Anterior_cingulate_and_paracingulate_gyri_Left

32 Anterior_cingulate_and_paracingulate_gyri_Right

33 Median_cingulate_and_paracingulate_gyri_Left

34 Median_cingulate_and_paracingulate_gyri_Right

35 Posterior_cingulate_gyrus_Left

36 Posterior_cingulate_gyrus_Right

37 Hippocampus_Left

38 Hippocampus_Right

39 ParaHippocampal_gyrus_Left

40 ParaHippocampal_gyrus_Right

41 Amygdala_Left

42 Amygdala_Right

43 Calcarine_fissure_and_surrounding_cortex_Left

44 Calcarine_fissure_and_surrounding_cortex_Right

45 Cuneus_Left

46 Cuneus_Right

47 Lingual_gyrus_Left

48 Lingual_gyrus_Right

49 Superior_occipital_gyrus_Left

50 Superior_occipital_gyrus_Right

51 Middle_occipital_gyrus_Left

52 Middle_occipital_gyrus_Right

53 Inferior_occipital_gyrus_Left

54 Inferior_occipital_gyrus_Right

55 Fusiform_gyrus_Left

56 Fusiform_gyrus_Right

57 Postcentral_gyrus_Left

58 Postcentral_gyrus_Right

59 Superior_parietal_gyrus_Left

60 Superior_parietal_gyrus_Right

61 Inferior_Parietal_gyrus_Left

62 Inferior_Parietal_gyrus_Right

63 SupraMarginal_gyrus_Left

64 SupraMarginal_gyrus_Right

65 Angular_gyrus_Left

66 Angular_gyrus_Right

67 Precuneus_Left

68 Precuneus_Right

69 Paracentral_Lobule_Left

70 Paracentral_Lobule_Right

71 Caudate_nucleus_Left

72 Caudate_nucleus_Right

73 Lenticular_nucleus,putamen_Left

74 Lenticular_nucleus,putamen_Right

75 Lenticular_nucleus,pallidum_Left

76 Lenticular_nucleus,pallidum_Right

77 Thalamus_Left

78 Thalamus_Right

79 Heschl_gyrus_Left

80 Heschl_gyrus_Right

81 Superior_temporal_gyrus_Left

82 Superior_temporal_gyrus_Right

83 Temporal_pole:superior_temporal_gyrus_Left

84 Temporal_pole:superior_temporal_gyrus_Right

85 Middle_temporal_gyrus_Left

86 Middle_temporal_gyrus_Right

87 Temporal_pole:middle_temporal_gyrus_Left

88 Temporal_pole:middle_temporal_gyrus_Right

89 Inferior_temporal_gyrus_Left

90 Inferior_temporal_gyrus_Right

61 Inf-Par-L

62 Inf-Par-R

63 Sup-Mar-L

64 Sup-Mar-R

65 Ang-Gyr-L

66 Ang-Gyr-R

67 Precun-L

68 Precun-R

69 Par-Lob-L

70 Par-Lob-R

71 Cau-Nuc-L

72 Cau-Nuc-R

73 Len-Nuc-L

74 Len-Nuc-R

75 Len-Nuc-L

76 Len-Nuc-R

77 Thala-L

78 Thala-R

79 Hes-Gyr-L

80 Hes-Gyr-R

81 Sup-Tem-L

82 Sup-Tem-R

83 TPol-S-L

84 TPol-S-R

85 Mid-Tem-L

86 Mid-Tem-R

87 TPol-M-L

88 TPol-M-R

89 Inf-Tem-L

90 Inf-Tem-R

91 Ce-Cru1-L

92 Ce-Cru1-R

93 Ce-Cru2-L

94 Ce-Cru2-R

95 Cere-3-L

96 Cere-3-R

97 Cere-4-5-L

98 Cere-4-5-R

99 Cere-6-L

100 Cere-6-R

101 Cere-7b-L

102 Cere-7b-R

103 Cere-8-L

104 Cere-8-R

105 Cere-9-L

106 Cere-9-R

107 Cere-10-L

108 Cere-10-R

109 Verm-1-2

110 Verm-3

111 Verm-4-5

112 Verm-6

113 Verm-7

114 Verm-8

115 Verm-9

116 Verm-10

91 Cerebelum_Crus1_Left

92 Cerebelum_Crus1_Right

93 Cerebelum_Crus2_Left

94 Cerebelum_Crus2_Right

95 Cerebelum_3_Left

96 Cerebelum_3_Right

97 Cerebelum_4_5_Left

98 Cerebelum_4_5_Right

99 Cerebelum_6_Left

100 Cerebelum_6_Right

101 Cerebelum_7b_Left

102 Cerebelum_7b_Right

103 Cerebelum_8_Left

104 Cerebelum_8_Right

105 Cerebelum_9_Left

106 Cerebelum_9_Right

107 Cerebelum_10_Left

108 Cerebelum_10_Right

109 Vermis_1_2

110 Vermis_3

111 Vermis_4_5

112 Vermis_6

113 Vermis_7

114 Vermis_8

115 Vermis_9

116 Vermis_10
